# Study on the Central Neural Pathways Connecting the Brain and Peripheral Acupoints Using Neural Tracers

**DOI:** 10.1111/cns.70554

**Published:** 2025-08-05

**Authors:** Junquan Liang, Weikang Sun, Yifu Zhou, Pan Zhang, Yuang Chen, Xuejie Li, Haoxuan He, Xiangkai Liu, Shibiao Zhou, Jingran Shen, Hongli Jiang, Yanzhang Chen, Rundong Tang, Luda Yan

**Affiliations:** ^1^ Shenzhen Bao'an Chinese Medicine Hospital The Seventh Clinical Medical School of Guangzhou University of Chinese Medicine Shenzhen Guangdong China; ^2^ The Brain Cognition and Brain Disease Institute (BCBDI), Shenzhen Institute of Advanced Technology (SIAT) Chinese Academy of Sciences (CAS), Shenzhen‐Hong Kong Institute of Brain Science‐Shenzhen Fundamental Research Institutions Shenzhen Guangdong China; ^3^ University of Chinese Academy of Sciences Beijing China; ^4^ School of Life Sciences Northwestern Polytechnical University Xi'an China; ^5^ Faculty of Healthy Science (FHS), Institute of Collaborative Innovation (ICI) University of Macau Macau China; ^6^ Beijing University of Chemical Technology Beijing China

**Keywords:** acupoints, neural projections, PRV‐CAG‐3 × mScarlet, retrograde tracing

## Abstract

**Background:**

Acupuncture is widely used for therapeutic purposes, but the neural mechanisms underlying its effects are not fully understood. This study aims to investigate the neural projections from acupoints and subcutaneous (sham acupoints) sites to the central nervous system, using retrograde tracing technology, to clarify the specificity of acupuncture's neural pathways.

**Methods:**

Adult C57BL/6J mice were injected with the retrograde tracer PRV‐CAG‐3 × mScarlet at seven acupoints (LI4, GV20, LI11, BL23, LR3, ST25, ST36) and their corresponding subcutaneous sites (sham acupoints). After 120 h, the brains were processed to assess viral expression and neural projections using histological analysis and imaging techniques.

**Results:**

The results revealed differences in neural projections between the acupoint groups and their corresponding subcutaneous sites. Acupoints exhibited common neural projections to regions such as the primary motor cortex (M1), secondary motor cortex (M2), gigantocellular reticular nucleus (Gi), and ventrolateral periaqueductal gray (VLPAG), while subcutaneous sites showed more diffuse and less specific projections.

**Conclusions:**

Compared to subcutaneous injection sites (sham acupoints), acupoints exhibit common neural projections in the brain. In contrast to the more diffuse neural projection patterns observed in subcutaneous sites (sham acupoints), acupoints display more numerous and specific neural projections in the brain.

## Graphical abstract

Retrograde viral tracing reveals that acupoints project to common and specific brain regions, while subcutaneous (sham) sites display more diffuse and inconsistent patterns, highlighting the neural specificity of acupuncture pathways.

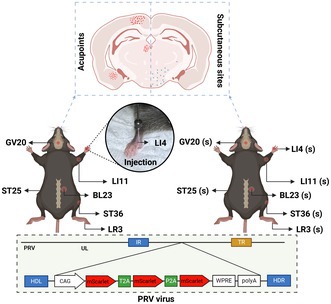

## 1 Introduction

The nervous system serves as the fundamental bridge connecting the brain with the peripheral body, enabling bidirectional communication through intricate neural networks [1]. As early as ancient Greece, Hippocrates (460–370 bce) recognized the brain as the central organ governing sensation, cognition, and movement. In *Sacred Disease* [2], he argued that “from the brain, and from the brain only, arise our pleasures, joys, laughter, and jests, as well as our sorrows, pains, griefs, and tears.” While the precise anatomical and physiological details were unknown in his time, his notion that the brain controls bodily functions laid the foundation for modern neuroscience.

Recent advancements in neuroanatomy and functional neuroimaging have provided empirical evidence supporting the intricate connectivity between peripheral sensory structures and central neural networks. Studies have shown that stimulation of specific acupuncture points can activate afferent neural pathways, modulating activity in brain regions such as the thalamus, somatosensory cortex, limbic system, and brainstem autonomic centers. For instance, functional magnetic resonance imaging (fMRI) studies have demonstrated that acupuncture at points like ST36 and LI4 is associated with activation of the hypothalamus and nucleus accumbens, as well as deactivation of the rostral anterior cingulate cortex, amygdala, and hippocampal complex [3].

However, the specific central neural pathways mediating this interaction remain incompletely understood. One promising approach to elucidating these pathways is the use of neural tracers, which allow for precise mapping of afferent and efferent neuronal circuits [4]. Previous studies have shown that peripheral acupuncture stimulation recruits sensory afferents such as Aδ and C fibers [5], which enter the spinal cord and ascend through the spinothalamic and spinoreticular tracts. Yet, the specific brainstem and supraspinal relay centers through which these signals are processed and integrated have not been fully delineated. Therefore, detailed tracing studies are needed to map the full trajectory of these signals from the periphery to their central targets.

In this study, we employed PRV‐based retrograde tracing, combined with classical neuroanatomical methods and advanced imaging, to systematically chart the central neural projections from seven commonly used acupuncture points. Our goal is to provide an anatomically detailed map of acupoint‐related central connections, offering structural insight into the neurobiological specificity of acupuncture.

## 2 Materials and Methods

### 2.1 Animals

Adult male C57BL/6J mice (20–25 g) were obtained from Sipeifu Biotechnology Co. Ltd. (Beijing, China). The animals were housed under standard laboratory conditions with a 12‐h light/dark cycle, temperature maintained at 22°C ± 2°C, and relative humidity at 50% ± 5%. Mice were provided with ad libitum access to standard laboratory chow and water.

All experimental procedures were conducted in accordance with the National Institutes of Health (NIH) Guide for the Care and Use of Laboratory Animals. Before the experiment, the animals were acclimated to the laboratory environment for at least 1 week, during which they were monitored daily for health status. Only animals showing no signs of illness, stress, or abnormal behavior were included in the study. The group size (*n* = 5) was selected based on previous similar tracer studies that have shown reliable and reproducible labeling with comparable numbers of animals. This design also aligns with ethical standards aiming to minimize animal usage while ensuring experimental validity.

To investigate the central neural pathways connecting the brain and peripheral acupuncture points [6], the mice were randomly divided into 14 groups, with five mice per group, comprising seven acupoint groups and their corresponding subcutaneous groups: (1) Shenshu (BL23) group and subcutaneous BL23 group; (2) Hegu (LI4) group and subcutaneous LI4 group; (3) Zusanli (ST36) group and subcutaneous ST36 group; (4) Quchi (LI11) group and subcutaneous LI11 group; (5) Tianshu (ST25) group and subcutaneous ST25 group; (6) Taichong (LR3) group and subcutaneous LR3 group; (7) Baihui (GV20) group and subcutaneous GV20 group.

To minimize experimental variability, all surgical and injection procedures were conducted at the same time of day. After the experimental procedures, the animals were humanely euthanized following ethical guidelines to minimize suffering.

### 2.2 Study Approval

All animal experiments and procedures were carried out in accordance with protocols approved by the ethics committee of the Shenzhen Institutes of Advanced Technology, Chinese Academy of Sciences (NO. YSB‐20231105‐LJS‐A1864).

### 2.3 Virus Construction

PRV‐CAG‐3 × mScarlet was generated based on the PRV‐Bartha strain using homologous recombination. BHK‐21 cells were cultured in DMEM supplemented with 10% fetal bovine serum (FBS) and incubated at 37°C in a 5% CO_2_ environment. When the cells reached 80% confluency, they were infected with PRV‐Bartha at a multiplicity of infection (MOI) of 0.01 and incubated for 1 h with gentle shaking every 15 min to ensure uniform infection. After 4–6 h post‐infection, cells were transfected with the donor plasmid HDL‐CAG‐3 × mScarlet‐HDR using Lipofectamine 2000 (Thermo Fisher Scientific) according to the manufacturer's protocol. The donor plasmid contained homology arms flanking the targeted insertion site in the PRV genome, allowing for homologous recombination. Fluorescent plaques were screened under an inverted fluorescence microscope at 48–72 h post‐transfection, and positive plaques were sequentially purified by limiting dilution and further passaged in BHK‐21 cells to obtain a stable recombinant virus stock. The final purified virus was amplified in BHK‐21 cells, and viral titers were determined using the TCID_50_ method, yielding a final concentration of 3 × 10^10^ PFU/mL.

### 2.4 Surgery and Viral Injections

Mice were anesthetized with an intraperitoneal injection of pentobarbital sodium (60 mg/kg) and secured in a stereotaxic apparatus (RWD, China). PRV‐CAG‐3 × mScarlet was injected into the designated acupoints and corresponding subcutaneous regions using a 10 μL syringe with a 33G needle (Neuros; Hamilton, Reno, USA) connected to a micro syringe pump (UMP3/Micro4, USA). Each site received 500 or 1000 nL of virus (3 × 10^10^ PFU/mL) at a rate of 500 nL/min (The specific injection volume and other parameters for each injection site are detailed in Table 1). The needle was retained in situ for an additional 10 min following injection to minimize reflux.

**TABLE 1:** The parameters for each injection site.

Injection site	Injection volume (μL)	Injection rate (nL/min)	Injection depth	Injection side (left/right)
BL23	1	50	2.5 (mm)	Right
BL23 (s)	1	50	Subcutaneous layer	Right
LI4	1	50	2 (mm)	Right
LI4 (s)	1	50	Subcutaneous layer	Right
ST36	1	50	2.5 (mm)	Right
ST36 (s)	1	50	Subcutaneous layer	Right
LI11	1	50	2.5 (mm)	Right
LI11 (s)	1	50	Subcutaneous layer	Right
ST25	1	50	2.0 (mm)	Right
ST25 (s)	1	50	Subcutaneous layer	Right
LR3	1	50	2.0 (mm)	Right
LR3 (s)	1	50	Subcutaneous layer	Right
GV20	0.5	50	Supraperiosteal layer^a^	—
GV20 (s)	0.5	50	Subperiosteal layer^a^	—

^a^ GV20 refers to a subperiosteal injection beneath the cranial periosteum, while GV20(s) indicates a supraperiosteal injection above the cranial periosteum. Although GV20(s) is anatomically distinct from other subcutaneous controls, it is categorized here as a subcutaneous (sham) control for consistency.

Following the procedure, mice were monitored for recovery. Tissue samples were collected at 120 h post‐injection for viral expression analysis, with all procedures conducted in a biosafety level‐2 (BSL‐2) laboratory under the approved protocols.

### 2.5 Perfusion, Section, and Imaging Examination

After the designated survival periods post‐injection, mice were deeply anesthetized and transcardially perfused with phosphate‐buffered saline (PBS), followed by 4% paraformaldehyde (PFA) in PBS to ensure proper fixation of tissues. Following perfusion, the brains were carefully extracted and post‐fixed in 4% PFA at 4°C for 24 h. Subsequently, the brain tissues were cryoprotected by immersion in 30% sucrose solution in PBS at 4°C until they sank, indicating complete infiltration. The brains were then embedded in an optimal cutting temperature (OCT) compound and rapidly frozen. Coronal sections of 30 μm thickness were cut using a cryostat (Leica CM1950, Germany) and collected onto glass slides. For imaging, the sections were scanned using the OLYMPUS VS200‐BU slide scanner, capturing high‐resolution images for subsequent analysis.

### 2.6 Data Presentation

To further illustrate the overlap and specificity of neural projections, we constructed network diagrams in the form of chord plots. These visualizations depict both the shared and unique brain regions identified between each acupoint and its corresponding subcutaneous (sham) injection group. Each chord represents a neural projection connection between the injection site and a labeled brain region. The directionality of projections is indicated by color‐coded lines: green for projections to the left hemisphere, blue for the right hemisphere, gray for bilateral projections (i.e., observed in both hemispheres), and purple for midline or non‐lateralized regions. This scheme allows for intuitive visualization of the laterality and distribution of neural pathways from each injection site. Chord plots were generated using custom R scripts with the circlize package, based on the qualitative classification of brain region labeling.

## 3 Results

**FIGURE 1:**
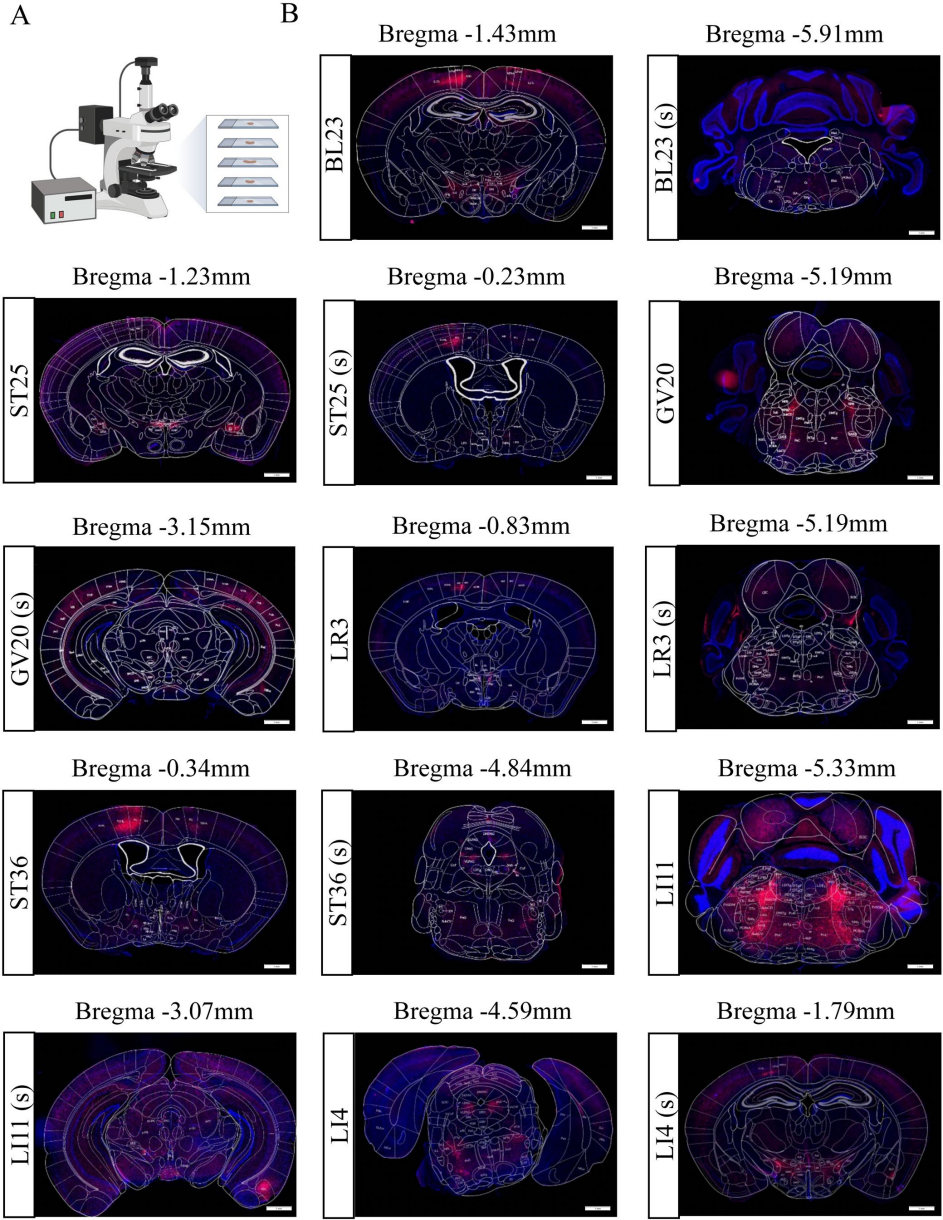
Representative images of viral expression in the seven acupoint groups and their corresponding subcutaneous (sham) groups. (A) Schematic of tissue section imaging. (B) Coronal brain sections from representative mice in each group, showing PRV‐CAG‐3 × mScarlet expression at selected Bregma levels. Acupoint and corresponding subcutaneous sites are shown in paired columns. Scale bars = 1 mm.

We examined neural projection patterns in seven acupoint groups and their corresponding subcutaneous (sham) control groups using PRV‐CAG‐3 × mScarlet retrograde tracing. Representative images of viral expression in these groups are provided in Figure 1.

### 3.1 Shared and Unique Brain Regions Among Acupoint Groups

Among the seven acupoint groups, four brain regions were consistently labeled across all groups: the gigantocellular reticular nucleus (Gi), the primary motor cortex (M1), the secondary motor cortex (M2), and the ventrolateral periaqueductal gray (VLPAG).

In addition to projections to these discrete nuclei, consistent labeling was also observed along the nigrostriatal bundle (ns), a major dopaminergic fiber tract. However, because it is a white matter pathway rather than a defined brain region, it was not included in the summary of shared brain regions.

**FIGURE 2:**
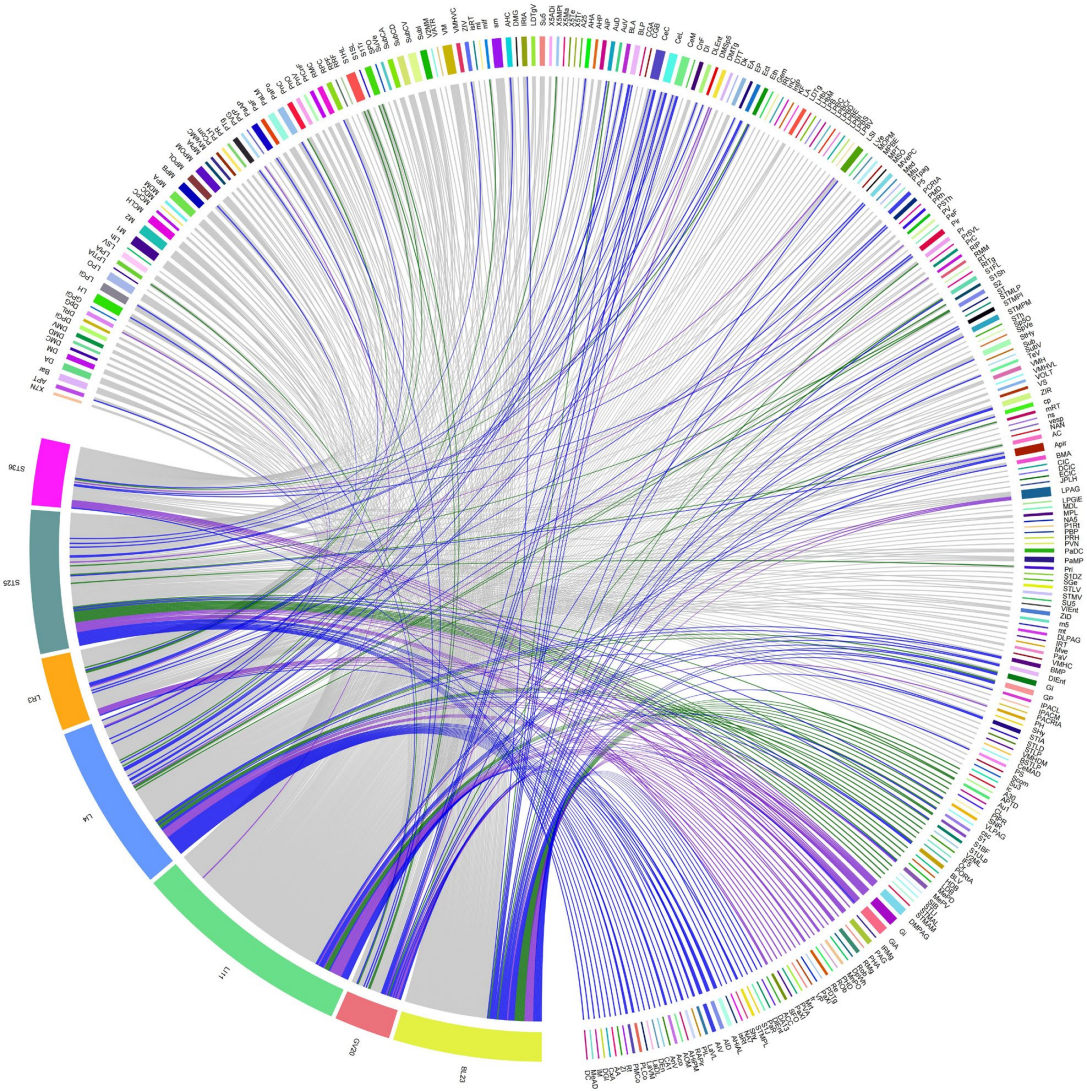
Network relationships of shared and unique brain regions among seven acupoint groups. (1) The left side of each diagram represents the injection sites, and the right side represents the corresponding brain regions with retrograde projections. (2) Each chord represents a projection connection. Color coding indicates hemisphere directionality: Green = left, blue = right, gray = bilateral, purple = undefined/non‐lateralized regions. (3) Some labeled targets are fiber tracts or undefined anatomical regions, included due to consistent retrograde labeling.

Each acupoint group also displayed distinct projection patterns, with LI4 showing 52 unique brain regions, GV20 showing 13, LI11 showing 77, BL23 showing 40, LR3 showing five, ST25 showing 39, and ST36 showing 10. Detailed information regarding the shared and unique brain regions is provided in Figure 2 and Table S1.

### 3.2 Shared and Unique Brain Regions Among Subcutaneous Groups

**FIGURE 3:**
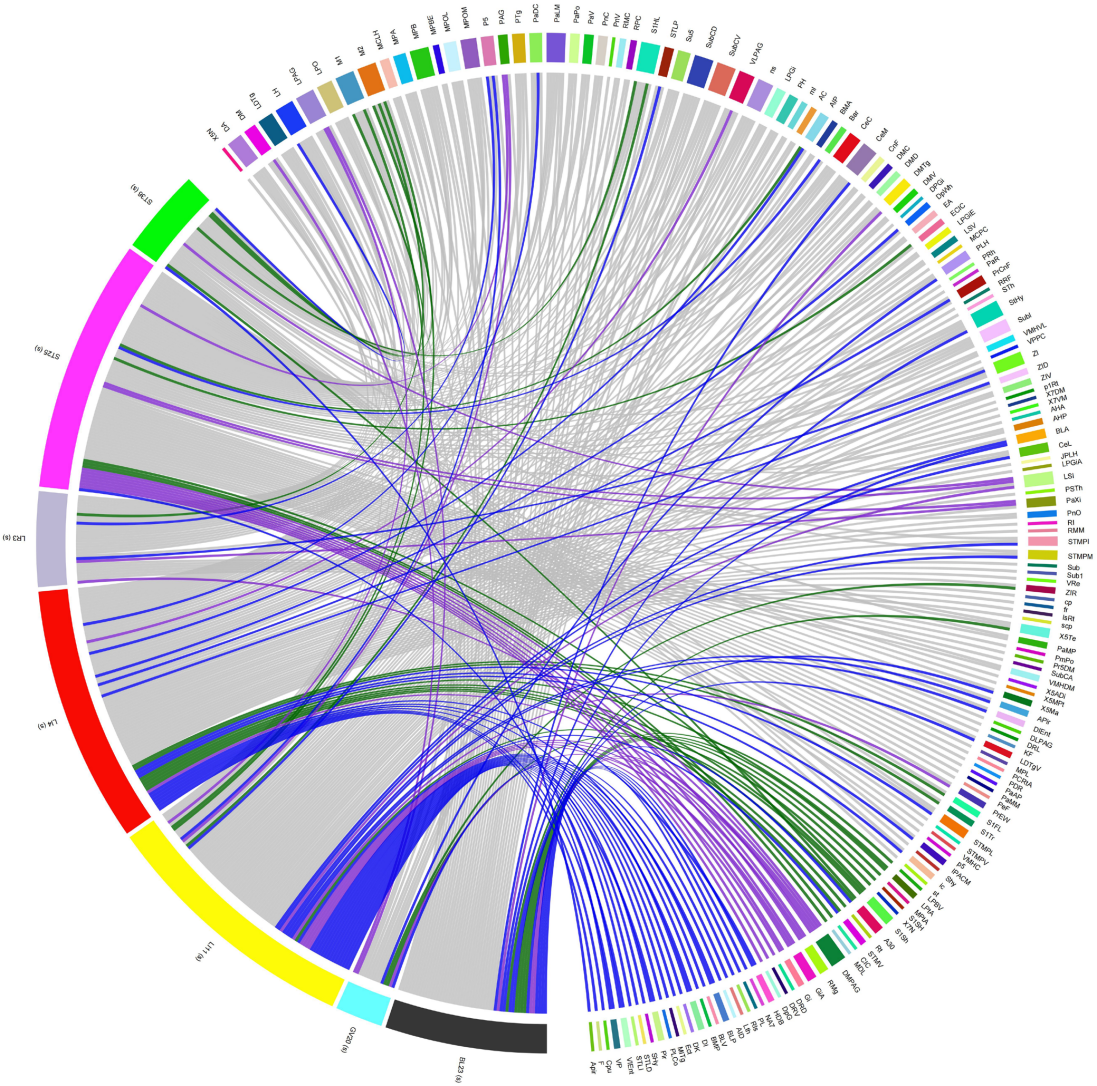
Network relationships of shared and unique brain regions among seven subcutaneous groups. (1) The left side of each diagram represents the injection sites, and the right side represents the corresponding brain regions with retrograde projections. (2) Each chord represents a projection connection. Color coding indicates hemisphere directionality: Green = left, blue = right, gray = bilateral, purple = undefined/non‐lateralized regions. (3) Some labeled targets are fiber tracts or undefined anatomical regions, included due to consistent retrograde labeling.

In the seven subcutaneous control groups, no shared brain regions of viral expression were observed. Each subcutaneous group showed unique viral labeling, with the LI4 group exhibiting 36 unique brain regions, the GV20 group showing five unique brain regions, the LI11 group showing 31 unique brain regions, the BL23 group showing 16 unique brain regions, the LR3 group showing four unique brain regions, the ST25 group showing 29 unique brain regions, and the ST36 group showing six unique brain regions. Detailed information regarding the unique brain regions for each subcutaneous group is provided in Figure 3 and Table S2.

### 3.3 Comparison Between Acupoint and Subcutaneous Groups

To systematically assess the differences in neural projections between acupoints and their corresponding subcutaneous (sham) sites, we analyzed the shared and unique brain regions across seven acupoint groups (LI4, GV20, LI11, BL23, LR3, ST25, and ST36) and their respective controls. The analysis focused on the distribution of PRV‐CAG‐3 × mScarlet labeling at 120 h post‐injection.

**FIGURE 4:**
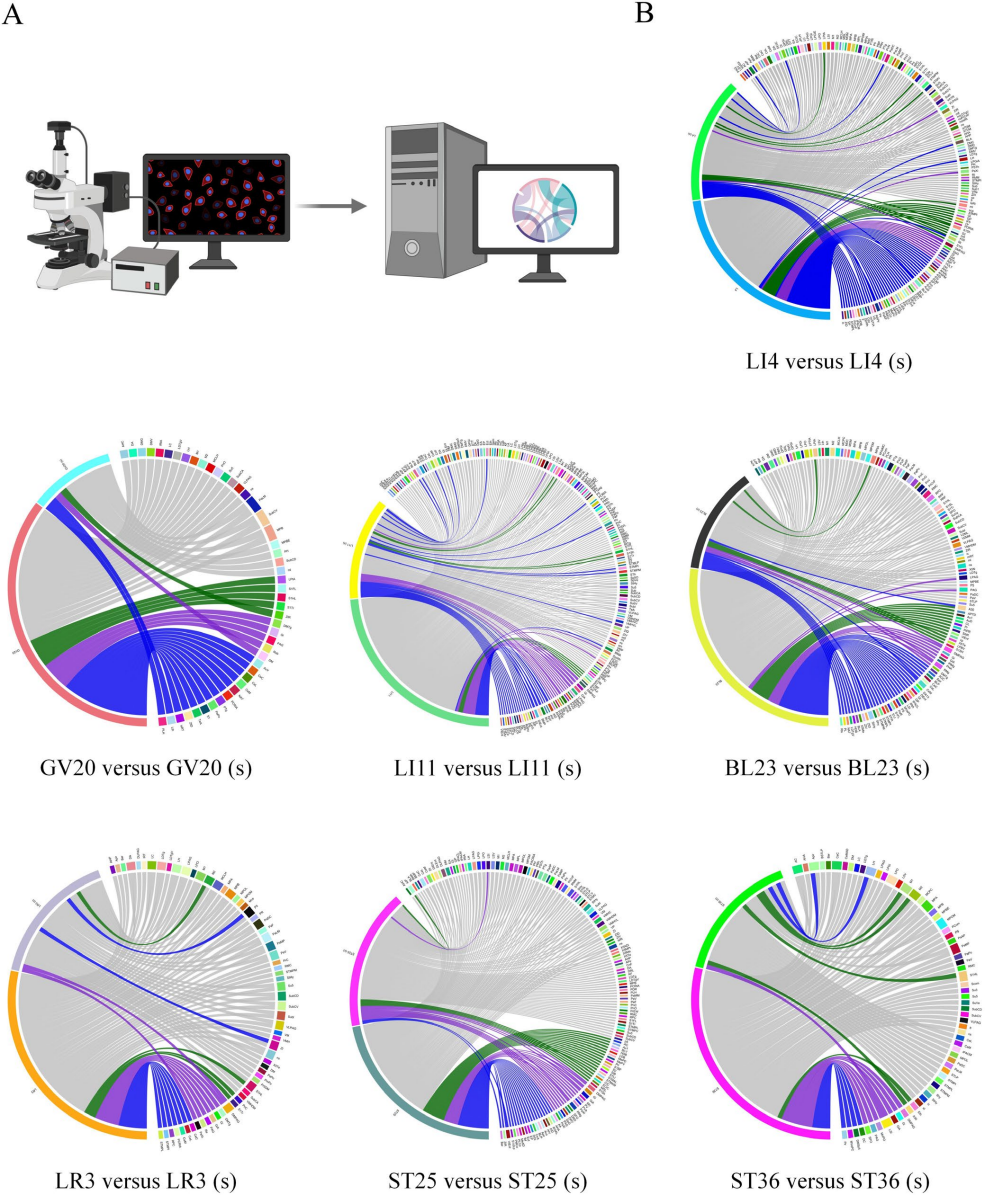
Network relationships of shared and unique brain regions between acupoint and subcutaneous groups. (A) Imaging and analysis schematic. (B) Network diagrams representing shared and unique brain region projections for each acupoint and its corresponding subcutaneous site. (1) The left side of each diagram represents the injection sites, and the right side represents the corresponding brain regions with retrograde projections. (2) Each chord represents a projection connection. Color coding indicates hemisphere directionality: Green = left, blue = right, gray = bilateral, purple = undefined/non‐lateralized regions. (3) Some labeled targets are fiber tracts or undefined anatomical regions, included due to consistent retrograde labeling.

Overall, acupoint injections consistently exhibited more numerous and more specific neural projections compared to subcutaneous injections. In all comparisons, the acupoint groups showed a higher number of unique brain regions, while the extent of shared regions varied depending on the injection site. Figure 4 presents the network relationships of shared and unique brain regions between each acupoint and its corresponding subcutaneous group, with detailed listings available in Table S3.

Specifically, the LI4 group exhibited 32 shared brain regions and 83 unique brain regions, compared to 36 unique regions observed in the subcutaneous LI4 group. Similarly, the GV20 group demonstrated only three shared brain regions, with 37 unique regions in the GV20 group and eight unique regions in its subcutaneous counterpart. The LI11 group showed 38 shared brain regions, accompanied by 106 unique regions in the acupoint group and 23 in the subcutaneous group. For the BL23 group, 25 brain regions were shared, while 81 and 19 unique regions were identified in the acupoint and subcutaneous groups, respectively. The LR3 group exhibited 18 shared brain regions, with 33 unique regions in the LR3 group and 8 in the subcutaneous group. In the ST25 group, 32 shared brain regions were identified, along with 70 unique regions in the acupoint group and 42 in the subcutaneous group. Lastly, the ST36 group presented 6 shared brain regions, with 39 unique regions in the acupoint group compared to 15 unique regions in the subcutaneous group.

These results indicate that acupoints not only project a greater number of distinct brain regions but also share specific neural pathways that are less evident following subcutaneous injections.

## 4 Discussion

### 4.1 Summary of Main Results

Acupuncture, as an important complementary and alternative therapy, is increasingly accepted worldwide due to its safety and effectiveness [7, 8]. Traditional acupuncture involves stimulating specific points known as acupoints, but the exact nature of these points and the distinction between acupoints and sham acupoints have been subjects of debate for centuries. Despite extensive discussion, no definitive conclusion has been reached [9]. In recent years, however, with continuous advancements in research—particularly the landmark studies [10] by Professor Ma's research team at Harvard University published in *Nature*—acupuncture has attracted increasing scientific interest. These studies have not only highlighted the potential of acupuncture but also paved the way for using advanced scientific techniques to investigate the anatomical basis of acupoints. In this context, we employed PRV virus retrograde tracing technology to explore the structural connections between peripheral acupoints (and sham acupoints) and the brain. Our findings uncovered several noteworthy neural projection patterns, which are discussed in the following sections.

#### 4.1.1 Shared Neural Projection Patterns in Seven Acupoint Groups, Absence of Common Projections in Seven Subcutaneous Sites

Our study reveals distinct differences in the neural projections from peripheral acupoints and subcutaneous (sham) sites to the central nervous system, providing valuable insight into the specificity of acupuncture's neural pathways. Across different acupoint groups, we observed both shared and unique projection targets in the brain.

We identified several common brain regions, including the Gi, M1, M2, and VLPAG, which consistently received retrograde projections from all acupoint groups. These areas are involved in essential functions such as motor control, pain modulation, autonomic regulation, and sensory processing [11, 12, 13]. The consistent neural projections to these regions suggest that acupuncture may influence fundamental brain circuits involved in pain modulation, motor control, and stress regulation, regardless of the specific acupoint used.

In addition to these shared regions, each acupoint group also showed unique neural projections to specific brain areas, highlighting the functional specificity of different acupuncture points. These differences in neural projections support traditional acupuncture theory, where each acupoint corresponds to different organs and systems, addressing specific physiological conditions. For example, neural projections from LI4 are connected to brain regions involved in pain and stress modulation [14], while projections from GV20 are associated with areas implicated in emotional regulation [15, 16].

Interestingly, our study also examined the effects of sham acupoints (subcutaneous sites). Despite being non‐specific, these sites still led to significant neural projections in the brain, particularly in regions involved in sensory and pain processing, such as the agranular insular cortex (AIP) [17], VLPAG [18, 19], and the peduncular lateral hypothalamus (PLH) [20]. These findings suggest that peripheral input from non‐specific sites can be transmitted to sensory‐related brain regions, which may underlie some of the observed therapeutic effects.

However, unlike the acupoint groups, the seven sham acupoints did not share common brain regions in their neural projections, indicating that each sham acupoint may have distinct and inconsistent projection patterns. This lack of convergence suggests that subcutaneous sites elicit more generalized and anatomically diffuse neural pathways, rather than specific or targeted projections. The absence of commonly projected brain regions further implies that peripheral input from non‐acupoint sites is processed in a less organized and site‐specific manner within the central nervous system.

In summary, although sham acupoints do not exhibit consistent or localized neural projection patterns, they still demonstrate distinct central pathways, which may partially account for their observable clinical effects. These findings underscore the widespread and non‐specific nature of peripheral input from non‐acupoint sites. In contrast, acupuncture at specific acupoints results in more organized and convergent neural projections, supporting the anatomical specificity of acupoints and reinforcing their potential relevance in targeted therapeutic applications.

#### 4.1.2 Differential Neural Projection Patterns Between Acupoints and Subcutaneous Sites (sham acupoints) in the Brain

The distinction between acupoints and sham acupoints (non‐acupoint or subcutaneous sites) has been a long‐standing debate in acupuncture research [21, 22]. Traditional acupuncture theory posits that each acupoint corresponds to specific physiological functions with distinct neural pathways and therapeutic effects. However, the concept of sham acupuncture or the use of non‐acupoint sites as controls in clinical and experimental studies has raised questions about whether acupuncture's effects are truly specific or if they could be attributed to generalized peripheral stimulation [23, 24].

Over the years, various studies have suggested that acupuncture at acupoints leads to specific brain activation patterns, while non‐specific stimulation, such as the use of sham acupoints, produces a more generalized effect [25, 26, 27]. This ongoing debate highlights the importance of investigating the anatomical specificity of the neural pathways associated with acupoints versus sham sites. Our study contributes to this field by directly comparing the central neural projections from seven representative acupoints and their corresponding sham sites using viral tracing techniques.

In our study, we compared the neural projections from several acupoints (LI4, GV20, LI11, BL23, LR3, ST25, and ST36) with their corresponding sham‐acupoint (subcutaneous) sites. The results revealed distinct differences in the neural projections between the acupoint and subcutaneous groups. For instance, LI4 showed neural projections to brain regions such as the periaqueductal gray (PAG), Gi, and posteromedial part of the amygdalohippocampal area (AHiPM), which are involved in pain modulation, motor control, and emotional regulation [28, 29], while LI4 (s) only had projections to regions like the forelimb area of the primary somatosensory cortex (S1FL), primarily related to basic sensory processing [30]. Similarly, GV20 demonstrated projections to regions linked to emotional regulation and cognitive processing [31, 32], including the PAG, M1, and S1, whereas GV20 (s) showed projections to fewer regions like Lth and PLH, which are more generalized. LI11 exhibited neural projections to areas involved in neuroendocrine function regulation and stress responses [33, 34], such as the PAG, perifornical nucleus (PeF), and median preoptic nucleus (MnPO), while LI11 (s) had more limited projections. In the cases of BL23, LR3, ST25, and ST36, neural projections were observed in brain regions associated with musculoskeletal health, autonomic regulation, and gastrointestinal function [35, 36, 37, 38, 39], while subcutaneous sites displayed more diffuse and less specific projections. These findings underscore the specificity of neural projections from acupoints, in contrast to the more generalized and less targeted projections seen with subcutaneous sites.

Our study provides evidence of distinct neural projection patterns between peripheral acupoints and subcutaneous sites (sham acupoints), supporting the ongoing debate in acupuncture research about whether there is a neural distinction between real and sham acupuncture points. We demonstrate that both real and sham acupoints have their own unique neural circuits, validating traditional acupuncture theory from a neurobiological perspective.

### 4.2 Limitations

While this study provides valuable insights into neural projections associated with acupoints, several limitations should be noted. First, the relatively small sample size warrants caution, and further studies with larger cohorts are needed to confirm the consistency and translational potential of these findings. Second, the use of animal models limits the direct extrapolation of these results to humans. Although many brain structures are conserved across species, interspecies differences in neuroanatomy and acupoint localization may affect the applicability of these findings to human acupuncture. However, rodent models remain essential for delineating fundamental neuroanatomical circuits and formulating hypotheses for clinical investigation. Lastly, the study focused on seven representative acupoints, and further research is needed to explore the neural correlates of a broader range of acupoints and their distinct functional pathways. Despite these limitations, this study provides a solid foundation for future research into the neurophysiological mechanisms underlying acupuncture, offering insights that could inform clinical research and therapeutic applications.

## 5 Conclusions

In this study, we investigated the neural projections associated with acupoints and subcutaneous (sham) sites, providing valuable insights into the specificity of acupuncture's neural pathways. Our findings lead to two key conclusions:

1. Compared to subcutaneous injection sites (sham acupoints), acupoints exhibit common neural projections in the brain.
  - Our results show that acupoints have shared neural projections that converge in specific brain regions. These shared projections reflect common neural circuits involved in essential physiological functions such as pain modulation, motor control, and stress regulation.
2. In contrast to the more diffuse neural projection patterns observed in subcutaneous sites (sham acupoints), acupoints display more numerous and specific neural projections in the brain.
  - Subcutaneous sites exhibited more diffuse and less consistent neural projections, whereas true acupoints showed more extensive and anatomically specific projections to defined brain regions. These differences underscore the structural specificity of neural pathways associated with acupoints compared to non‐acupoint sites. Further studies are warranted to refine the mapping of these projections and to elucidate the central networks potentially underlying the effects of acupuncture.

## Author Contributions

Conceptualization: Junquan Liang. Data curation: Xuejie Li, Haoxuan He, Xiangkai Liu, Shibiao Zhou, Jingran Shen. Formal analysis: Junquan Liang, Yifu Zhou, Pan Zhang, Yuang Chen. Methodology: Junquan Liang, Xuejie Li, Haoxuan He, Xiangkai Liu, Shibiao Zhou, Jingran Shen. Project administration: Junquan Liang, Hongli Jiang. Resources: Junquan Liang, Rundong Tang. Software: Junquan Liang, Luda Yan, Yanzhang Chen. Visualization: Junquan Liang, Weikang Sun, Yuang Chen. Writing – original draft: Junquan Liang. Writing – review and editing: Junquan Liang.

## Ethics Statement

The animal experiments were approved by the Ethics Committee of the Shenzhen Institutes of Advanced Technology, Chinese Academy of Sciences (Approval No. YSB‐20231105‐LJS‐A1864).

## Conflicts of Interest

The authors declare no conflicts of interest.

## Supporting information


**Tables S1–S4:** Brain region abbreviations used in the manuscript.

## Data Availability

The data that support the findings of this study are available from the corresponding author upon reasonable request.
