# Supplementary material for: Study on the Central Neural Pathways Connecting the Brain and Peripheral Acupoints Using Neural Tracers
Source: CNS Neurosci Ther. 2025 Aug 5;31(8):e70554. doi: 10.1111/cns.70554 (PMC12322693; doi:10.1111/cns.70554)
Supplement: Supplementary file 1 — Tables S1–S4: Brain region abbreviations used in the manuscript. [file CNS-31-e70554-s001.docx]

**Supplement Table 1. Shared and unique brain regions of viral expression in acupoint groups**

| **Injection site** | **Unique brain regions** | **Shared brain regions** |
| --- | --- | --- |
| LI4 | JPLH (B), VOLT, IF5 (L), AHiPM (R), S1DZ (B), 5MPt (L), PVN (B), PDTg, MDL (B), m5 (L), ECIC (B), PORtA (L), RIP, VATR (B), VS (R), PBP (B), LaVM (R), GI (L), csc, AHC (R), AuD (R), Rt (R), AOM (R), P1Rt (B), SGe (B), mlf (B), DCIC (B), PaXi, GP (L), CA1 (R), PRH (B), Or (L), LPGiE (B), BLA (R), MePV (R), DpG, LaDL (R), AnV (R), LH (R), Eth (R), SU5 (B), DLEnt (R), MPOL (R), DEn (R), ml (L), NA5 (B), Pri (B), APT (R), Subl, S1Sh (L), PaAP (R), CIC (B) | Gi, M1 (B), M2 (B), VLPAG (B), ns (B) |
| GV20 | PaPo (R), S1 (R), PTg (R), Rob, isRT (R), DMG (B), S1FL (L), NA7 (R), LPtA (L), LPGi (R), GiA (R), AuV (R), TeA (R) |  |
| LI11 | A25 (B), AuD (B), LA (B), BMA (R), S1Sh (B), Eth (B), vesp (B), PeF (B), P1pag (B), RtTg (B), m5 (B), ST (B), Ect (B), LPBV (B), IntP (B), PVG, BLP (B), InC (B), Dk (B), MPT (B), 5Ma (B), CGA (B), IRt (B), mt (B), MVePC (B), RT (B), PMD (B), LHbM (B), STMPI (B), LPBCr (B), MSO (B), Sp5O (B), LVe (B), PH, CnF (B), CGB (B), S1 (L), Gem (B), PCRtA (B), LPB (B), SpVe (B), TeV (L), sm (B), Pr5VL (B), KF (B), S2 (B), 5MPt (B), RIP (B), Bilateral, Sub (B), PIL (R), SubV (B), LPBC (B), EP (B), S1FL (B), DpWh, LPBI (B), LHbL (B), Rob, STh (B), Med (B), DMSp5 (B), PHD, TeA (B), LPBD (B), MOPM (B), AuV (B), LPBS (B), Mtu (B), S1ULp (L), 5Tr (B), STMLP (B), LPBE (B), VMHVC (B), 5Te (B), MePV, PV (B) |  |
| BL23 | MDC (B), S1FL (R), DlEnt (R), ml (B), Shy (R), VIEnt (L), IRMg, RRF (B), APTD (L), SNR (L), DA13 (R), Lth (B), StHy (R), PtPR (L), IPACL (R), PVP (B), A30 (L), V2ML (B), Pr (L), ZIV (R), cp (L), S1SL (B), IRtA (R), PS (R), LSI (R), PHA, ic (B), AuD (L), GPGi (B), LPTtA (B), SPO (B), S1J (R), STMPM (R), RPF (B), ACC (R), CL (L), PVG (B), mRT (B), V2MM (B), PaR (R) |  |
| LR3 | Mve (B), IRT (B), VMH (B), RPC, Right PnC (L) |  |
| ST25 | Mrt, IPACL (B), EP (R), VMHC (B), DIEnt (B), PACRtA (B), STLD (B), STIA (B), HDB (L), ZIR (R), MePD (L), AHA (R), S1ULp (R), IPACM (B), BMP (B), AA (R), LDB (L), Pri (L), GI (B), STMAM (L), PVA, CxA (R), PaXI, DGI (R), BLV (L), PV, MeAD (R), MePV (L), GP (B), STLP (B), SHy (B), VIEnt (R), STMAL (L), PH (B), AC (L), STLI (L), SIB (L), IM (R) |  |
| ST36 | CeMAD (B), Eth (L), DMSp5 (R), LSI, Scom (B), BSTLP (B), Su3 (B), fr (B), DC (R), PS (B) |  |

**Note:** (1) Directional classification refers to the left (L), right (R), bilateral (B), or non-lateralized regions. (2) Brain region localization was determined according to the 4th edition of *Paxinos and Franklin's Mouse Brain in Stereotaxic Coordinates*. (3) Some labeled targets are fiber tracts or undefined anatomical regions, included due to consistent retrograde labeling.

**Supplement Table 2. Shared and unique brain regions of viral expression in subcutaneous groups**

| **Injection site** | **Unique brain regions** | **Shared brain regions** |
| --- | --- | --- |
| LI4 (s) | PrCnF (R), JPLH (B), STMV (L), STMPL (R), ic (R), CeC (R), BLA (B), ZID (R), PaXi (B), DpWh (R), LSI (B), RMM (B), 7DM (B), LPGiA (B), S1FL (L), VLPAG, PSTh (B), lsRt (B), S1Tr (L), Cpu (R), fr (B), Sub (B), F (R), 7VM (B), Rt (L), LSV, IPACM (R), RI (B), AIP (R), PaDC (R), cp (B), VRe (B), ZIR (B), Sub1 (B), AHA (B), scp (B) | **/** |
| GV20 (s) | 7N (L), PLH (R), DM, ZIR (L), Lth (R) |  |
| LI11 (s) | STh (B), VPPC (B), ECIC (B), SHy (R), STLP (R), RRF (B), MCPC (B), CeL (R), Bar (B), BLV (R), AIP (B), AID (R), PaR (B), DIEnt (R), PRh (B), PLCo (R), STLD (R), STLI (R), MITg (R), PrEW, DK (R), DPGi (B), STMV (R), APir (R), ZID (B), STMPM (R), DpWh (B), BMP (R), Ect (R), STMPI (R), p1Rt (B) |  |
| BL23 (s) | 5N (B), LPBV (L), Rls (R), PAG (B), LDTgV (R), 5Te (L), PL (R), StHy (R), NA7 (R), 5Ma (R), S1SH (L), A30 (R), PnV (B), MPtA (L), CeM (R), DMTg |  |
| LR3 (s) | Pr5DM (B), SubCA (B), ZI (R), PmPo (B) |  |
| ST25 (s) | 5Ma (B), AIP (L), DLPAG (B), DRV, VMHC (B), DIEnt (B), p5 (B), PDR (B), 5ADi (B), PaMM (B), KF (B), PeF (B), APir (B), 5MPt (B), MPL (B), DpG, DRL (B), PaAP (B), DRD, STMPV (B), S1FL (B), CIC (L), LDTgV (B), Bar (R), S1Tr (B), PrEW (B), PCRtA (B), MDL (L), ECIC (L) |  |

**Note:** (1) Directional classification refers to the left (L), right (R), bilateral (B), or non-lateralized regions. (2) Brain region localization was determined according to the 4th edition of *Paxinos and Franklin's Mouse Brain in Stereotaxic Coordinates*. (3) Some labeled targets are fiber tracts or undefined anatomical regions, included due to consistent retrograde labeling.

**Supplement Table 3. Shared and unique brain regions of viral expression between acupoint and subcutaneous groups**

| **Injection site** | **Unique brain regions** | **Shared brain regions** |
| --- | --- | --- |
| LI4 versus LI4 (s) | **LI4:** 5ADi (B), 5MPt (L), AC (B), AHC (R), AHiAL (R), AHiPM (R), AID (R), AIV (R), AOM (R), APT (R), Aco (R), AnV (R), Apir (B), AuD (R), BLV (R), BMP (R), Bar (B), CA1 (R), CIC (B), DCIC (B), DEn (R), DIEnt (R), DLEnt (R), DPGi (B), DRL (B), DTT (B), DpG, ECIC (B), Ect (R), Eth (R), GI (L), GP (L), Gi, GiA, IF5 (L), LC (B), LDTgV, LPGi (B), LaDL (R), LaVL (R), LaVM (R), MCLH (B), MDL (B), MDM (B), MPL (B), MPOL (R), MPtA (B), MePV (R), NA5 (B), Or (L), P1Rt (B), PAG, PBP (B), PDTg, PLCo (R), PMCo (R), PORtA (L), PRH (B), PVN (B), PaAP (R), PaMP (B), PnV (B), Pr (B), Pri (B), RAPir (R), RIP, RPC (B), S1DZ (B), S2 (L), SGe (B), STIA, STLV (B), SU5 (B), SubCA (B), Subl (B), VATR (B), VOLT, VS (R), csc, isRt, m5 (L), mlf (B)  **LI4 (s):** 7DM (B), 7VM (B), A30 (L),AHA (B), AHP (B), Cpu (R), DMC (B), DMD (B), DMTg (B), DMV (B), DpWh, F (R), HDB (R), IPACM (R), LDTg (B), LPGiA (B), LSV, PH (B), PSTh (B), RI (B), RMM (B), S1FL (L),STMPI (B), STMPL (R), StHy (B), Sub (B), Sub1 (B), VP (R), VRe (B), ZID (R), ZIV (B), cp (B), fr (B), ic (R), lsRt (B), scp (B) | ns (B), M2 (B), LPAG (B), CeC (B), PTg (B), EA (B), LPGiE (B), PaLM (B), SubCV (B), M1 (B), BLP (R), CeM (B), JPLH (B), PnC (B), S1Sh (L), (B), P5 (B), SubCD (B), Subl (B), LSI (B), Pir (R), MPB (B), DA (B), ZIR (B), BMA (B), LPO (B), MPA (B), CeL (B), MPOM (B), PnO (B), STMPM (B), S1HL (B) |
| GV20 versus GV20 (s) | **GV20:** AHC (B), AuV (R), CeC (R), CeL (R), CeM (R), DA (B), DMG (B), DMTg, DMV (B), Gi, IRtA (B), LC (B), LDTgV (B), LH (B), LPtA (L), M1 (B), M2 (B), MCLH (B), NA7 (R), PAG , PCRtA (R), PTg (R), PaPo (R), PnO (B), Rob, S1 (R), S1FL (L), S1HL (L), S1Tr (L), Su5 (B), SubCA (B), TeA (R), VLPAG (B), ZID (R), isRT (R), ns (B)  **GV20 (s):** DM, Lth (R), MPBE (B), PH (B), PLH (R), SubCD (B), ZIR (L), ml (B) | PaLM (B), SubCV (B), MPB (B) |
| LI11 versus LI11 (s) | **LI11:** 5ADi (B), 5MPt (B), 5Ma (B), 5Te (B), 5Tr (B), A25 (B), AHA (B), AHC (B), AHP (B), AHiAL (R), AIV (R), APT (B), Apir (R), AuD (B), AuV (B), CGA (B), CGB (B), DLEnt (B), DM (B), DMSp5 (B), DTT (B), Dk (B), DpG (B), EP (B), Eth (B), GI (R), GP (R), Gem (B), IRt (B), IRtA (B), InC (B), IntP (B), KF (B), LA (B), LHbL (B), LHbM (B), LPB (B), LPBC (B), LPBCr (B), LPBD (B), LPBE (B), LPBI (B), LPBS (B), LPBV (B), LVe (B), LaVL (R), MCLH (B), MOPM (B), MPA (B), MPBE (B), MPT (B), MSO (B), MVeMC (B), MVePC (B), MePV, Med (B), MnPO, Mtu (B), P1pag (B), PCRtA (B), PHD, PIL (R), PMD (B), PSTh (B), PV (B), PVG, PaAP (B), PaF (B), PaPo (B), PeF (B), PnC (B), Pr (B), Pr5VL (B), PrC (B)  **LI11 (s):** AC (B), APir (R), DK (R), ECIC (B), LPGiE (B), MCPC (B), MITg (R), PAG, PLCo (R), PTg (B), PaR (B), PrCnF (B), PrEW RRF (B), SHy (R), STLD (R), STLI (R), STLP (R), STMV (R), VIEnt (R), VPPC (B), ZI (B), p1Rt (B) | ns (B), LC (B), CeC (B), VLPAG (B), StHy (B), ZIV (B), CnF (B), AID (R), ZID (B), BMP (R), Su5 (B), EA (B), PLH (B), VMHVL (B), SubCV (B), CeM (B), DMD (B), LPGi (B), BLV (R), LSV (B), SubCD (B), Subl (B), AIP (B), DIEnt (R), MPB (B), DA (B), DPGi (B), LDTg (B), DMTg (B), STh (B), DMV (B), LH (B), PRh (B), Bar (B), MPOL (B), MPOM (B), LPO (B), DMC (B) |
| BL23 versus BL23 (s) | **BL23:** 7N (B), ACC (R), APT (B), APTD (L), Apir (R), Au1 (L), AuD (L), BLP (R), Bar (B), CL (L), CeC (R), CeL (R), DA13 (R), DMC (B), DMD (B), DMV (B), DPGi (B), DRL (B), DlEnt (R), DpG (B), GI (R), GP, GPGi (B), Gi, GiA, IPACL (R), IRMg IRtA (R), LPGi (B), LPTtA (B), LSI (R), LSV (B), Lth (B), MCPC (B), MDC (B), MDM (B), MVeMC (B), MVePC (R), PCom (B), PHA, PLH (B), PR (B), PS (R), PVG (B), PVP (B), PaAP (B), PaF (B), PaR (R), PnO (B), Pr (L), PrCnF (B), PtPR (L), RPF (B), RRF (B), S1FL (R), S1J, S1SL (B), S1Tr (B), SNR (L), SPO (B), STIA (R), STMPI (R), STMPL (R), STMPM (R), Shy (R), SuVe (B), SubCA (B), Subl (B), V2ML (B), V2MM (B), VIEnt (L), VMHDM (B), ZID (R), ZIR (B), ZIV (R), cp (L), ic (B), mRT (B), ml (B)  **BL23 (s):** 5Ma (R), 5N (B), 5Te (L), BLA (R), DMTg, HDB (R), LDTg (B), LDTgV (R), LPBV (L), MPBE (B), NA7 (R), P5 (B), PL (R), PaDC (B), PaV (B), Rls (R), S1SH (L), STLP (B), Su5 (B) | ns (B), M2 (B), RMC (B), PTg (B), VLPAG (B), StHy (R), MCLH (B), DM (B), PaLM (B), SubCV (B), M1 (B), CeM (R), PnC (B), PnV (B), SubCD (B), PaPo (B), MPB (B), DA (B), RPC (B), MPOL (B), LH (B), MPA (B), LPO (B), MPOM (B), S1HL (B) |
| LR3 versus LR3 (s) | **LR3:** AHP (B), Apir (B), Bar (B), CeC (R), CeL (R), CeM (R), DLPAG (B), DMTg, Gi, GiA, IRT (B), LDTgV (B), LPO (B), MPA (B), MPOL (B), MPOM (B), Mve (B), PAG, PCRtA (R), PR (B), PaF (B), PnC (L), PrC (B), RMC (B), RPC (R), Re, S1Tr (L), STMPI (R), STMPL (R), STMPM (B), StHy (B), VM (B), VMH (B)  **LR3 (s):** 5Te (B), DM (B), PaPo (B), PaXi, PmPo (B), Pr5DM (B), SubCA (B), VMHDM (B) | ns (B), LPAG (B), LC (B), VLPAG (B), MCLH (B), PaV (B), Su5 (B), PaLM (B), SubCV (B), M1 (B), PaDC (B), SubCD (B), Subl (B), MPB (B), DA (B), LDTg (B), LH (B), PaMP (B) |
| ST25 versus ST25 (s) | **ST25:** AA (R), AC (L), AHA (R), AHC (B), AHiAL (R), AID (R), AIV (R), Aco (R), Apir (R), Au1 (L), BLA (B), BLP (R), BLV (L), BMA (B), BMP (B), CeL (B), CxA (R), DGI (R), DI (B), DLEnt (B), EA (B), EP (R), Ect (R), GI (B), GP (B), HDB (L), IM (R), IPACL (B), IPACM (B), IRtA (B), LDB (L), MeAD (R), MePD (L), MePV (L), Mrt, PACRtA (B), PH (B), PLCo (R), PMCo (R), PRh (B), PSTh (B), PV, PVA, PaDC (B), PaMP (B), PaXI, Pir (B), Pri (L), RMM (B), S1BF (L), S1ULp (R), S2 (L), SFO, SHy (B), SIB (L), STIA (B), STLD (B), STLI (L), STLP (B), STLV (B), STMAL (L), STMAM (L), STMV (B), VM (B), VP (B), ZID (B), ZIR (R), ZIV (B), isRt (B)  **ST25 (s):** 5ADi (B), 5MPt (B), 5Ma (B), 5Te (B), AHP (B), APir (B), Bar (R), CIC (L), CnF (B), DA (B), DRD, DRL (B), DRV, DpG, ECIC (L), KF (B), LDTg (B), LDTgV (B), MDL (L), MPB (B), PAG, PCRtA (B), PDR (B), PLH (B), PaMM (B), PaV (B), PaXi, PeF (B), PnC (B), PnO (B), PrEW (B), RMC (B), RPC (B), S1FL (B), S1Sh (L), S1Tr (B), STMPL (B), STMPV (B), Su5 (B), SubCD (B), SubCV (B), p5 (B) | ns (B), LPAG (B), M2 (B), VIEnt (R), CeC (B), VLPAG (B), StHy (B), PTg (B), PrCnF (B), MCLH (B), DM (B), MPL (B), VMHVL (B), PaLM (B), M1 (B), CeM (B), LPGi (B), ZI (B), VMHC (B), LSV (B), Subl (B), PaPo (B), DIEnt (B), DLPAG (B), PaAP (B), MPOM (B), MPOL (B), LH (B), MPA (B), LPO (B), VMHDM (B), S1HL (B) |
| ST36 versus ST36 (s) | **ST36:** AHA (B), BSTLP (B), Bar (B), CeMAD (B), DC (R), DM (B), DMPAG, DMSp5 (R), Eth (L), Gi, GiA, LC (B), LDTg (B), LPAG (B), LPGi (B), LSV (B), MCPC (B), MPB (B), MPBE (B), MVePC (R), MnPO, PAG, PCom (B), PS (B), PaAP (B), PaPo (B), PaV (B), Pir (R), RMC (B), SFO, Scom (B), Su3 (B), Su5 (B), SuVe (B), SubCD (B), SubCV (B), VLPAG (B), fr (B), ns (B)  **ST36 (s):** A30 (L), CeL (B), CeM (B), IPACM (B), MPOL (B), PaDC (B), PaLM (B), STLP (B), STMPI (B), STMPL (B), STMPM (B), Shy (B), StHy (B), ic (B), st (B) | CeC (B), MPOM (B), MPA (B), LPO (B), PaMP (B), AC (B) |

**Note:** (1) Directional classification refers to the left (L), right (R), bilateral (B), or non-lateralized regions. (2) Brain region localization was determined according to the 4th edition of *Paxinos and Franklin's Mouse Brain in Stereotaxic Coordinates*. (3) Some labeled targets are fiber tracts or undefined anatomical regions, included due to consistent retrograde labeling.

**Supplementary Table 4. Brain region abbreviations used in the manuscript**

| **Abbreviation** | **Full Name** |
| --- | --- |
| Gi | Gigantocellular reticular nucleus |
| M1 | Primary motor cortex |
| M2 | secondary motor cortex |
| ns | nigrostriatal bundle |
| VLPAG | Ventrolateral periaqueductal gray |
| AIP | agranular insular cortex |
| PLH | peduncular lateral hypothalamus |
| PAG | periaqueductal gray |
| AHiPM | posteromedial part of the amygdalohippocampal area |
| PeF | perifornical nucleus |
| MnPO | median preoptic nucleus |
| S1FL | Forelimb area of primary somatosensory cortex |
| PAG | Periaqueductal gray |

**Note:** Brain region localization was determined according to the 4th edition of *Paxinos and Franklin's Mouse Brain in Stereotaxic Coordinates*.
